# To Match or Not to Match in Epidemiological Studies—Same Outcome but Less Power

**DOI:** 10.3390/ijerph7010325

**Published:** 2010-01-26

**Authors:** Tomas Faresjö, Åshild Faresjö

**Affiliations:** Department of Medical and Health Sciences, Community Medicine, Linköping University, SE-58183 Linköping, Sweden; E-Mail: Ashild.Olsen.Faresjo@Liu.se

**Keywords:** epidemiology, matching, case-control study, gender

## Abstract

This study aimed to analyze the possible resemblance or difference in outcome in a case-control study of quality of life for IBS patients compared to controls free from the disease, when a matching procedure for age and sex was applied for the control group compared to when all participating subjects were included in the control group. The main result was that almost the same and identical results were found irrespective of whether matching or not matching was applied in this epidemiological case-control study. The matching procedure however, slightly diminished the statistical power of the results.

## 1. Introduction

Matching is not uncommon in epidemiological studies and refers to the selection of unexposed subjects’ *i.e*., controls that in certain important characteristics are identical to cases. Most frequently matching is used in case-control studies but it can also be used in cohort studies. The matching procedure is often directed towards classical background factors such as sex and age. If data collection from subjects is expensive, it is desirable to optimize the amount of information obtained per subject. This could be done by matching controls with cases [1,2]. The method of matching controls with identified cases is widespread in a wide range of epidemiological studies such as studies of cardiovascular diseases [3], cancers [4–6], pediatrics [7], gastroenterology [8–10], and surgery [11].

Generally, to match controls with cases is an often applied device to control for confounding in studies. This is especially relevant when there is a substantial difference in the occurrence of possible confounders between cases and controls. A confounder could be defined as a third variable that is both associated to the independent and the dependent variable. The existence of a confounder introduces a bias since an observed effect could be attributed to the confounder rather than the studied independent variable. A misconception concerning matching is that its purpose is to raise the validity of the study. However, the role of matching is not to increase validity, but rather to increase the efficiency of a study. The improvement in efficiency by matching is generally small unless the variable is a strong confounder [12].

In a systematic literature review of techniques used to measure influences of confounding in observational studies of health effects of drug therapies 29 studies were analysed. Of these, almost all used regression techniques as the main method to control for confounding. Some studies also used stratification and only four used matching to address confounding [13]. In most studies regression-like techniques are routinely used for adjustment for confounding but more empirical evaluations comparing these methods in different situations are needed [14].

So the fundamental methodological question with general implications for the design of epidemiological studies is if one should match or not match controls with cases. What methodological benefits or drawbacks could be made by matching procedures? The aim of this study was to analyze the possible resemblance or difference in results and outcome in a case-control study when a matching procedure for age and sex was applied for the control group compared to when all participating subjects were included in the control group.

## 2. Methods

### 2.1. Study Design

The data analyzed in this study is based on a case-control design measuring quality of life for individuals in the age-group 18–65 years diagnosed during a 5-year period with the gastrointestinal disorder IBS (ICD-10 K-58 p). The IBS patients were identified from computer-based medical records from three Swedish primary health care centers in the city of Linköping (135,000 inhabitants) located in the south east of Sweden. The primary health care centers covered in total a catchment population of 40,000 inhabitants and hosted practically all general practitioner consultations in their respective geographical areas.

### 2.2 Data Collection

An analysis of the computerized medical records at the primary health care centers identified 487 IBS patients in the age-group 18–65 years. The collection of baseline data from the IBS patients has been described elsewhere [9,16]. Through the local census population register, a control population of 4,427 individuals in the same age group (18–65 years) was randomly selected from the same geographical area as the identified IBS cases. Prior to the survey, a check was made to ensure that the individuals in the control population did not have any registered IBS diagnosis during the period studied.

### 2.3. Questionnaire

A postal questionnaire was sent to both the cases and controls. The main part of this questionnaire was the generic health-related quality of life measure Short Form 36 (SF-36). This well established instrument includes eight multi-item scales measuring the extent to which an individual’s health limits his or her physical, emotional and social functioning. All questions were asked in respect to the previous four weeks. Responses in the SF-36 were transferred to a standard scale, ranging from 0 (the worst possible score) to 100 (the best possible score) [15]. The questionnaire also included some demographic data. The response rate in the survey for the IBS patients was 71.3% and for the controls 61.6% thus leaving a group of n = 347 cases and a population of n = 2,727 un-matched controls. The overall response rate in the study was 62.6 %.

### 2.4. The Matching Techniques

The matching technique applied in this study was based on the variables age and sex. Three controls per IBS case were randomly selected and matched by age and sex from the control population (n = 347 cases and n = 1,041 controls). For the un-matched analyze, the final study population comprised the number of responders of the postal questionnaire, n = 347 IBS cases and n = 2,727 controls.

### 2.5. Statistical Analyses

All data were stored in a common database and statistically analyzed using the SPSS version 15.0 program (SPSS Inc., Chicago, IL, USA). The significance of differences between cases and the controls were calculated by chi^2^ for background variables and for the SF-36 scale by ANOVA tests, 95% confidence intervals were also calculated. A p-value of p < 0.05 was considered statistically significant. The study was approved by the Research Ethics Committee at Linkoping University, Sweden.

## 3. Results

The proportion of females was significantly (p < 0.0001) higher for the IBS cases and also accordingly among the matched controls in comparison with the un-matched controls from the general population. For the other background variables like age-groups, civil status and educational level there were no significant difference between cases, matched controls or un-matched controls, see Table 1.

In Table 2 the mean scores of quality of life for the IBS-cases was compared to their sex and age-matched controls. A general result was that quality of life was significantly lower on all eight SF-36 dimensions for the IBS-cases than among their matched controls. For males these differences was not statistically significant for the dimensions; physical functioning, physical role and emotional role.

In Table 3 the mean scores for SF-36 for the IBS-cases was compared to an un-matched population from the general population. Also when quality of life scores for IBS-cases were compared with a larger but un-matched control group their quality of life was significantly lower on all eight dimensions. Also in this analysis, using un-matched controls, male IBS-cases and controls were not statistically significant different for the dimensions; physical functioning, physical role and emotional role. However, the level of significance for males was higher when the IBS-cases were compared to un-matched controls.

## 4. Discussion

A main finding in this study was that almost the same and identical main results were found irrespectively of matching or not matching was applied. However, the resemblance between matched and un-matched females was more in concordance than the same results for males. This is most likely an effect of increased statistical power related to population size when the larger un-matched group was used as comparison. A principal way in epidemiological studies to increase precision, reduce random error and increase the statistical power of a study is to enlarge the number of subjects [2].

The role of matching in epidemiological research is somewhat controversial. Many epidemiologists routinely match on age and sex, even when they are not regarded as confounders nor extremely distributed. This practice is questionable since a matched case-control study nevertheless often requires complex statistical analysis. Further the matching procedure might reduce the statistical power in the study, which also was shown in the results of this study. But, on the other hand if the distribution of the matching factor in the case group is distributed in an extremely way, matching could be a reasonable option. A general perception of matching procedures in epidemiological studies is that it controls for confounding. Another way to control for the influence of confounding factors like age and sex than matching is to apply multivariate analysis [16]. The multivariate analysis makes it also possible to increase the power of the study since all controls could be included in the analysis.

## 5. Methodological Considerations

A possible study limitation is that the matched and un-matched data used in this study was already collected so the analyze was based on a watching technique. In this study, females constitute two third (n = 251) of all n = 347 identified cases of IBS, thus in this respect the males are a minority group. The overload of females with IBS in this study is quite in common with other epidemiological findings of this type of gastrointestinal disease [17–19]. The relatively minor male group gives a somewhat more fluctuating pattern of results for males than for females when matching or not matching procedures was applied. However, differences in quality of life outcome that were statistically significant for males were still statistically significant no matter matching or not matching were used.

## 6. Conclusions

This study shows that almost the same and identical results were found irrespective of matching or not matching was applied in this epidemiological case-control study. The matching procedure however, slightly diminished the statistical power of the results, but only for males since this group only represented one third of all the cases. This tendency that the statistical power will be reduced when matching procedures is applied is a circumstance that talks against matching in epidemiological studies. In most epidemiological studies the procedure of multivariate analysis is instead preferable to handle confounding situations in the analysis.

## Figures and Tables

**Table 1.** Socio-demographic data for IBS cases (n = 347) and sex and age-matched controls (n = 1,041) and all controls (n = 2,727).

	**IBS cases n = 347**		**Matched control group n = 1,041**		**All controls n = 2,727**	
	**n**	**%**	**n**	**%**	**n**	**%**
**Sex:** Male Female	96 251	27.7 72.3	288 753	27.7 72.3	1,309 1,418	48.0 52.0***
**Age groups:** 15 to 24 25 to 44 45 to 64	36 147 164	10.4 42.4 47.3	108 441 492	10.4 42.4 47.3	428 1,167 1,132	15.7 42.8 41.5
**Civil status:** Living alone Married/cohabitant Divorced Widow/widower	56 258 26 4	16.3 75.0 7.6 1.2	160 787 65 18	15.5 76.4 6.3 1.7	528 1,989 153 31	19.5 73.6 5.7 1.1
**Educational level:** Primary school (low) Secondary school Upper secondary school University college or university (high)	57 60 80 146	16.6 17.5 23.3 42.6	149 200 229 453	14.5 19.4 22.2 43.9	362 524 701 1,115	13.4 19.4 25.9 41.3

*** p < 0.0001 compared to cases and matched controls.

**Table 2.** Mean SF-36 scores (95% CI) for IBS cases (n = 347) and sex and age-matched controls (n = 1,041).

	**Female**		**Male**		**Total**	
**SF-36 scale:**	**IBS cases (n = 251)**	**Matched controls (n = 753)**	**IBS cases (n = 96)**	**Matched controls (n = 288)**	**IBS cases (n = 347)**	**Matched controls (n = 1,041)**
**Physical functioning**	84.2 (81.6 to 86.4)	89.0 ** (87.7 to 90.3)	89.3 (86.1 to 93.0)	92.1 n.s (90.4 to 94.0)	86.0 (83.5 to 88.1)	90.0 *** (89.0 to 91.0)
**Physical role**	71.0 (66.3 to 75.6)	84.0 *** (81.3 to 86.0)	82.1 (76.0 to 89.0)	88.0 n.s (85.0 to 91.1)	74.1 (70.0 to 78.0)	85.1 *** (83.0 to 87.0)
**Bodily pain**	68.0 (65.0 to 71.0)	80.0 *** (78.4 to 81.5)	74.2 (69.3 to 79.1)	83.0 ** (80.5 to 85.1)	69.4 (67.1 to 72.1)	81.1 *** (79.5 to 82.0)
**General health**	62.0 (59.0 to 65.1)	75.0 *** (73.4 to 76.4)	67.4 (63.0 to 72.1)	75.0 ** (73.0 to 77.2)	63.5 (61.1 to 66.1)	75.0 *** (74.1 to 76.2)
**Vitality**	50.0 (47.0 to 53.8)	62.0 *** (60.0 to 63.2)	59.0 (54.2 to 63.4)	66.5 * (64.1 to 69.1)	52.3 (50.0 to 55.1)	63.1 *** (62.0 to 64.3)
**Social functioning**	75.1 (72.0 to 78.4)	87.0 *** (85.0 to 88.1)	83.0 (77.5 to 87.8)	89.0 * (86.5 to 91.1)	77.0 (74.2 to 80.0)	87.3 *** (86.1 to 89.0)
**Emotional role**	69.5 (65.0 to 75.0)	84.2 *** (82.0 to 86.3)	86.0 (76.1 to 90.1)	87.0 n.s (86.1 to 92.1)	73.2 (69.1 to 77.2)	85.4 *** (84.0 to 87.2)
**Mental health**	67.0 (64.3 to 69.5)	77.0 *** (76.1 to 78.2)	74.3 (71.1 to 78.1)	79.0 * (76.4 to 81.0)	69.0 (67.1 to 71.0)	77.4 *** (76.4 to 79.0)

*** p < 0.0001,

** p = 0.001,

* p < 0.05, n.s = not significant.

**Table 3.** Mean SF-36 scores (95% C.I.) for IBS cases (n = 347) and the total un-matched control group (n = 2,727).

	**Female**		**Male**		**Total**	
**SF-36 scale:**	**IBS cases (n = 251)**	**Un-matched controls (n = 1,418)**	**IBS cases (n = 96)**	**Un-matched controls (n = 1,309)**	**IBS cases (n = 347)**	**Un-matched controls (n = 2,727)**
**Physical functioning**	84.2 (81.6 to 86.4)	89.7 ** (88.8 to 90.5)	89.3 (86.1 to 93.0)	93.0 n.s (92.3 to 94.0)	86.0 (83.5 to 88.1)	91.3*** (91.0 to 92.0)
**Physical role**	71.0 (66.3 to 75.6)	84.0 *** (82.5 to 86.0)	82.1 (76.0 to 89.0)	88.1 n.s (87.1 to 90.0)	74.1 (70.0 to 78.0)	86.1*** (85.0 to 87.1)
**Bodily pain**	68.0 (65.0 to 71.0)	80.6 *** (79.5 to 81.7)	74.2 (69.3 to 79.1)	84.4*** (83.3 to 85.5)	69.4 (67.1 to 72.1)	82.4*** (82.1 to 83.2)
**General health**	62.0 (59.0 to 65.1)	75.0 *** (74.0 to 76.1)	67.4 (63.0 to 72.1)	77.1 *** (76.1 to 78.1)	63.5 (61.1 to 66.1)	76.1*** (75.2 to 77.0)
**Vitality**	50.0 (47.0 to 53.8)	62.0 *** (61.0 to 63.0)	59.0 (54.2 to 63.3)	67.2** (66.1 to 68.4)	52.3 (50.0 to 55.1)	64.4*** (64.0 to 65.2)
**Social functioning**	75.1 (72.0 to 78.4)	87.0 *** (86.0 to 88.1)	83.0 (77.5 to 87.8)	90.1** (89.0 to 91.0)	77.0 (74.2 to 80.0)	88.3*** (87.5 to 89.0)
**Emotional role**	69.5 (65.0 to 75.0)	83.0 *** (81.1 to 84.5)	83.0 (76.1 to 90.1)	87.0 n.s (85.1 to 88.2)	73.2 (69.1 to 77.2)	85.0*** (84.0 to 86.0)
**Mental health**	67.0 (64.3 to 69.5)	76.0 *** (75.1 to 77.0)	74.3 (71.1 to 78.1)	79.4** (78.5 to 80.4)	69.0 (67.0 to 71.0)	78.0*** (77.0 to78.3)

*** p < 0.0001,

** p = 0.001,

* p < 0.05, n.s = not significant.
